# Taste loss in cancer patients: clinicians’ perceptions of educational materials and diagnostic tools

**DOI:** 10.1007/s00520-023-07794-4

**Published:** 2023-05-24

**Authors:** Lakmani Tharaka Galaniha, Alissa A. Nolden

**Affiliations:** Department of Food Science, University of Massachusetts, Amherst, MA 01003 USA

**Keywords:** Taste alterations, Clinician perception, Dysgeusia, Flavor, Cancer

## Abstract

**Purpose:**

Cancer therapy is essential and lifesaving; however, it can have short- and long-term consequences on patients’ health. Up to 87% of cancer patients report changes in taste function, yet patients report a lack of support from clinicians regarding their experience with taste loss during and following treatment. Thus, the objective of this study was to assess clinicians’ knowledge and experience with managing patients with taste loss and identify potential gaps in the availability of educational materials and diagnostic tools.

**Method:**

In an online survey, sixty-seven participants who identify as clinicians and practice in the United States and work with cancer patients that complain of taste problems answered questions on their knowledge and experience supporting cancer patients experiencing changes in taste function and provided their opinion on access to educational materials.

**Results:**

The current study reports gaps in participants’ knowledge of taste and taste disorder terminology, with 15.4% correctly defining both taste and flavor and roughly half were familiar with specific taste disorder classifications. Over half of the participants reported not having access to adequate information to help their patients manage taste alterations. Only two-thirds of participants reported routinely asking patients if they are experiencing changes in taste function.

**Conclusion:**

Clinicians’ responses emphasized the need to improve access to educational materials regarding taste changes and increase the availability of information regarding management strategies. Addressing these inequities in education and improving the standard of care is the first step in improving the care for cancer patients suffering from altered taste function.

## Introduction

There were 18.1 million new cancer cases in the United States in 2018, and it is estimated that by 2040 new cancer cases per year will increase to 29.5 million [1]. Improvements in care and advances in treatment have helped to improve the survival rates, and it is expected that by 2030 there will be 22.2 million cancer survivors in the US [1]. While cancer treatment is prescribed to be lifesaving, individuals can experience unpleasant and long-lasting side effects. These side effects vary in frequency, duration, and severity and can negatively impact the quality of life.

One prevalent side effect is changes in taste function. According to self-reported data, up to 93% of cancer patients report experiencing changes in taste perception [2–6]. The sense of taste refers to the perception of five qualities (sweet, salt, sour, bitter, and umami) derived from the chemical stimulation of taste receptors in the tongue, pharynx, larynx, and soft palate [7]. While taste and flavor are terms used interchangeably, they are distinct terms, with flavor referring to the combination of sensations from the gustatory, olfactory, chemesthesis, and somatosensory systems. These systems are separate sensory pathways with distinct peripheral and central neural mechanisms [8]. While gustation and olfaction have distinct mechanisms, patients and consumers alike do not segment these sensations but rather describe their experience. For example, patients may describe their experience or symptoms as a “change in food flavor.” Similarly, smell loss can present itself as a reduction in food flavor; and it is only through clinical assessments do clinicians uncover the root cause for their complaints related to food flavor. Uncovering the specific sensory symptom is important for directing clinical support and treatment strategies. While both taste and smell symptoms are both prevalent among cancer patients, the present investigation is focused on taste as Nolden and colleagues (2018) identified that taste, rather than smell function, was associated with changes in food intake.

Loss or changes to taste perception can profoundly impact dietary intake, treatment outcome, and daily life. Among cancer patients, complaints of changes in taste function are associated with gastrointestinal problems (e.g., loss of appetite, constipation, diarrhea, abdominal cramp, nausea, and vomiting) [3, 9], oral issues (e.g., oral pain, dry mouth, and oral mucositis) [3, 10, 11], food-related problems (e.g., secondary anorexia, food aversions, weight loss, and malnutrition), compromised immunity, and reduced treatment outcomes [12–14]. Together, these data and others demonstrate that altered taste perception is associated with lower quality of life due to its impact on physical and psychosocial dimensions [2–5, 9, 15–18]. While more research is needed, these data support the theoretical framework that taste function may be necessary for identifying patients at greater risk for nutritional impact symptoms, gastrointestinal problems, quality of life, and treatment outcome.

There are different types of taste disorders, all falling under the classification of dysgeusia, the change in gustatory function. According to published literature, individuals experiencing taste disorders can be further classified as having reduced taste sensitivity (hypogeusia), enhanced taste sensitivity (hypergusia), complete loss of taste (ageusia), a persistent presence of an unpleasant sensation like bitter or metallic (palinageusia), or perception of a taste in the absence of a stimulus (phantageusia) [15, 19–21]. To clinically diagnose these taste disorders, an objective test is considered the gold standard; however, most cancer-related taste assessments are evaluated using self-report. Among cancer patients, dysgeusia, generally described in the literature as changes in taste, is estimated to be between 17.6 and 93% [2–6, 15, 22]. Several reviews have highlighted the variability in the reported prevalence of dysgeusia in cancer patients, mainly attributing it to the different methodologies used to evaluate taste function (e.g., self-reported vs. clinical assessment) and variability in clinical characteristics (e.g., cancer type and treatment) [23, 24]. Another challenge is deciphering patient complaints (e.g., tastes bad) and translating these descriptions that use terminology we use in everyday life, which can be challenging to untangle. For example, the interchangeable nature of the use of terms taste and flavor makes it challenging for patients and clinicians to communicate and assess problems with taste perception [7,25,26]. This challenge was also evident during the COVID-19 pandemic as noted by Parma and colleagues, as patients and consumers experience foods and beverages wholistically, rather than perceiving taste and smell in isolation.

Despite the high prevalence and adverse effects of altered taste function, clinicians often overlook taste-related symptoms as they are non-life threatening [22]. Due to the lack or limited support from clinicians, with few to no options for treatments or management strategies, cancer patients describe adjusting their lives to cope with taste alterations on their own. In some cases, cancer patients describe taste loss as an unavoidable side effect of having cancer or cancer treatment [5, 16, 27]. The combination of losing their sense of taste and lack of clinical support is detrimental to their quality of life, causing many negative emotions, including disappointment, frustration, and sadness, and interferes with their daily rituals, especially around dining events and roles with their family members [2, 16, 23, 28].

There is a need to improve clinical support for cancer patients suffering from taste loss. Currently, there is a limited understanding of clinicians’ familiarity and knowledge of taste alterations. Therefore, this study aims to evaluate clinicians’ familiarity and knowledge of terminologies and methods for assessing and diagnosing gustatory function. This information can be used to inform and develop targeted training and educational material for clinicians to better support cancer patients suffering from changes in taste function.

## Methods

### Survey participants and procedures

In an online platform, this study targeted clinicians who work with cancer patients that complain of taste problems. Potential participants were recruited through online social media networking websites, professional networks, and posting advertisements in hospitals. As an incentive, participants had the option to enter a raffle drawing to win one of ten commercial vouchers. To be eligible, participants must be at least 18 years old, reside in the U.S., and work with cancer patients that complain of taste problems. The survey ran between January 2020 and February 2021. This online survey received approval from The Institutional Review Board at The University of Massachusetts. The online survey was launched using Compusense Cloud software (Guelf, Canada).

### Questionnaire

Upon obtaining informed consent, participants answered questions related to demographics, including age, gender, and ethnicity, followed by questions related to profession and professional experience, including the number of years in practice and specific cancer types of the patients they work with. For the present analysis, this study investigates participants’ responses to a variety of questions pertaining to two main themes (Table 1). The first theme focuses on assessing clinicians’ knowledge of taste-related terminologies and taste assessment tools. The second theme considers clinicians’ experience with educational materials to learn about taste alterations.

**Table 1:** Questions assessing clinicians’ knowledge of (1) taste terminology and assessment tools and (2) experience with educational materials

	Questions
1^st^ theme	▪ From the list of options provided, select the best definition for “taste.” ▪ From the list of options provided, select the best definition for “flavor.” ▪ Are you familiar with the term “dysgeusia”? ▪ From the list of options provided, select the best definition for “dysgeusia.” ▪ How would you identify whether a patient is suffering from dysgeusia? ▪ Do you evaluate your patients routinely for taste alterations or ask them whether they experience taste changes? ▪ Select all of the methods you use to evaluate taste alterations. ▪ How challenging are these assessment tools to use in a clinical setting? ▪ From a list of taste disorders, select which ones you have heard of. ▪ For each taste disorder, match the definition.
2^nd^ theme	▪ Do you think you have access to adequate information in order to help patients manage their taste problems? If no, explain. ▪ In your opinion, what are the most useful educational materials or supportive information available for clinicians to learn about taste alterations (e.g., a specific website, American Cancer Society, professional societies, and training programs for clinicians)? ▪ Do you have access to these materials? If no, explain. ▪ How challenging you find to access and use educational materials? ▪ What are the most important criteria that an improved education tool should consist of to enable learning about taste alterations? ▪ Upon the development of tools for treating/evaluating patients with taste problems, what do you believe are the biggest and immediate needs?

*The taste alteration terms hypergeusia, hypogeusia, phantageusia, ageusia, palinageusia, and caogeusia were given in boxes, and the definitions for each taste alteration term were given separately

### Statistical analysis

Descriptive statistics were performed to report and analyze the frequency of clinicians’ responses using R Studio (version 3.6.2). Open-ended questions were coded and analyzed for themes using NVivo 12 Plus (version 12.6.0).

## Results

### Participant characteristics

Of the 76 individuals that consented to participate, 71 completed the questionnaire. Four participants were ineligible as they indicated they did not work with patients complaining of taste problems. The final analysis includes 67 participants, of which the majority were female (74.6%), white (74.6%), and belonged to the age category 25–34 years old (58.2%). In terms of profession, most participants were otolaryngologists (34.3%) and speech pathologists (28.4%) and have been practicing for less than 10 years (61.2%). Table 2 provides a summary of the demographic information of the participant population.

**Table 2:** General characteristics of participants

	*n*	*%*
Gender		
Female	50	74.6
Male	17	25.4
Age (years)		
25–34	39	58.2
35–44	14	20.9
45–54	2	3.0
55–64	7	10.5
65<	5	7.5
Ethnicity		
Asian	10	15.0
Black or African American	1	1.5
Hispanic or Latino	2	3.0
White	50	74.6
Other	4	6.0
Profession		
Dietitian	8	11.9
Nurse	5	7.5
Oncologist	2	3.0
Otolaryngologist	23	34.3
Speech pathologist	19	28.4
Other	10	14.9
Years practicing		
1–10	41	61.2
11–20	15	22.4
21–30	3	4.5
31–40	7	10.4
41–50	1	1.5

### *1*^*st*^*theme:assessment of clinicians’knowledge of taste-related terminologies and taste assessment tools*

The questions in the first theme assessed clinicians’ knowledge of taste-related terminologies and familiarity and experience with taste assessment tools.

#### Disparities in participants’ knowledge of taste, flavor, and taste disorders

To determine participants’ familiarity with taste and flavor, participants were asked to select the correct definition for taste and flavor from a list of possible definitions. Roughly half (51.9%) of participants selected the correct definition for taste, with 30.8% selecting the correct definition for flavor. For flavor, 40.4% selected the definition of taste. Only 15.4% of participants selected the correct definitions for taste and flavor. Conversely, 1.9% reported “I don’t know” for both the taste and flavor definitions.

To assess knowledge and familiarity with taste disorders, participants were first asked if they were familiar with the term “dysgeusia,” with 96.2% indicating that they were familiar with the term. When asked to select the correct definition, 98% selected the correct definition as an altered, impaired, distorted, or abnormal taste perception. Participants were asked to indicate if they had heard of a specific type of taste disorder, followed by selecting the definition for each taste disorder. The taste disorder that was most familiar to participants was ageusia 61.5%, followed by hypogeusia (57.7%), hypergeusia (51.9%), and phantageusia (25%). Very few participants were familiar with caogeusia (1.9%), and no participants were familiar with palinageusia. When the participants were asked to select the definition for each taste disorder, most participants were able to correctly define hypogeusia (94.2%), ageusia (92.3%), hypergeusia (86.5%), and phantageusia (84.6%). In comparison, only 26.9% selected the correct definition for palinageusia, and no participants correctly defined caogeusia.

#### Methods to assess taste function

**Fig. 1:**
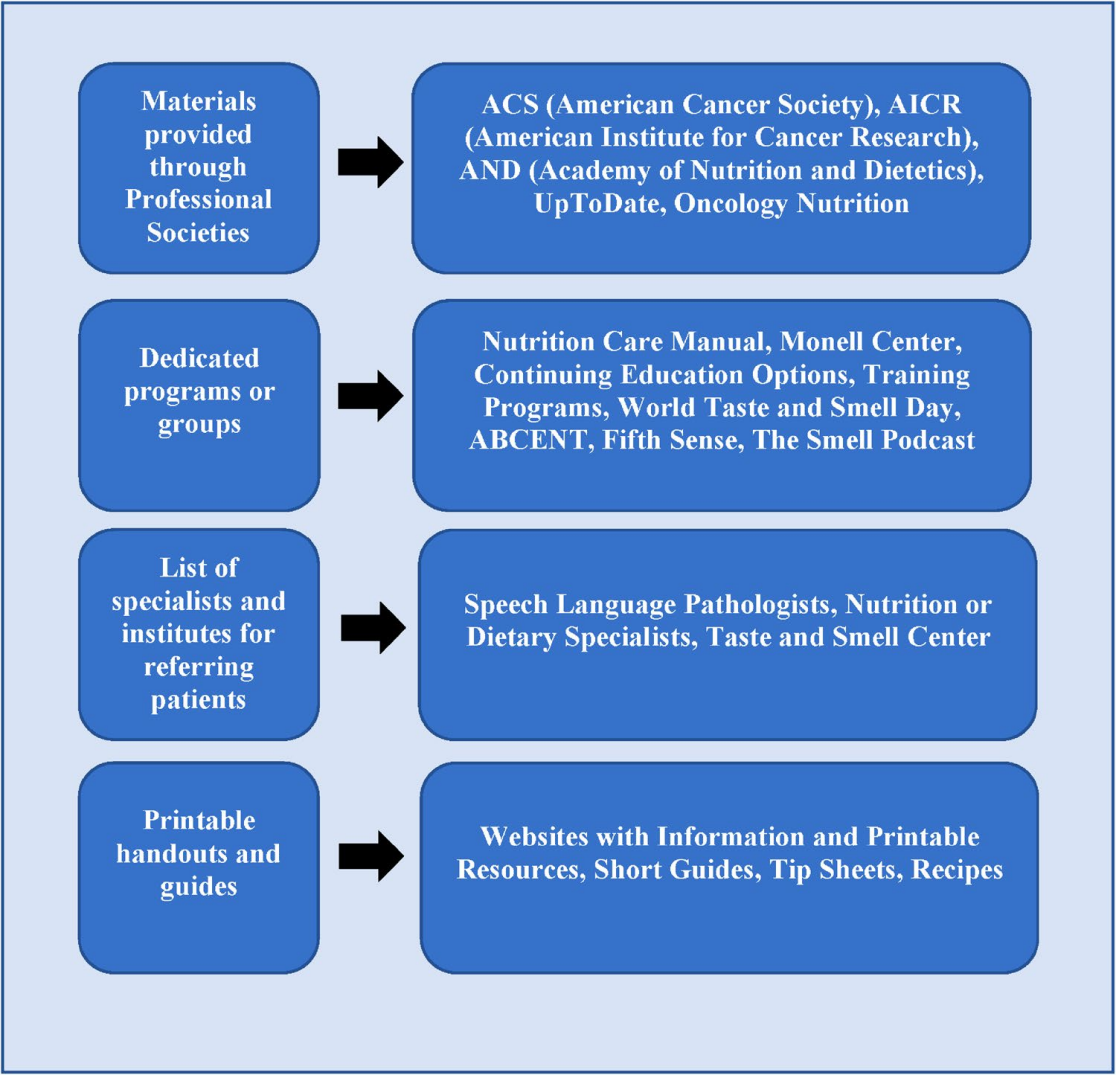
Participants’ comments on potential resources for clinicians to help patients with managing taste alterations

Participants were asked if they routinely evaluate their patients for taste alterations to assess the clinical taste function evaluation methods. Roughly two-thirds of participants (64.4%) reported that they routinely evaluate or ask their patients if they are experiencing changes in taste function. Of these participants, 18.6% reported using self-assessment questionnaires, 5.1% reported evaluating taste intensity responsiveness (i.e., suprathreshold response, recording the intensity response for different concentrations of taste solutions (e.g., sucrose in water)), and 1.7% reported measuring recognition threshold levels (i.e., the lowest concentration at which a taste can be detected) (Fig. 1A). Roughly half (49%) of the participants reported asking or assessing patients for taste function indicated using other methods not listed. However, upon examining text descriptions, many listed or described self-assessment methods. Therefore, it is assumed that these participants were familiar with assessment tools but were unsure of the type of assessment (i.e., self-assessment vs. psychophysical evaluation).

In a similar line of questioning, participants were asked about their familiarity with assessment tools for diagnosing dysgeusia. Participants were asked to select all the methods they have used to identify whether a patient is suffering from dysgeusia. The most common selection was diagnosing dysgeusia by self-assessments or questionnaires (61.5%), while roughly one quarter (22%) indicated using psychophysical methods for assessing taste function. As a follow-up, participants were asked to select all the psychophysical methods that they have used to evaluate dysgeusia, revealing that 21.2% of participants have assessed taste intensity responsiveness, with fewer participants assessing recognition threshold levels (11.5%), detection threshold levels (5.8%), gustatory evoked potential levels (5.8%), and electrogustometry (1.9%).

To assess the perceived ease of use of assessment tools (e.g., self-assessment questionnaires and psychophysical evaluation), participants rated how challenging it is to employ taste assessment tools on a 5-point scale ranging from “not at all” to “very” challenging. For taste assessment tools, most reported that they were “somewhat challenging” (40.7%), followed by “quite a bit” (22%) and “a little bit” (17%), while 6.8% indicated “very” challenging. However, 13.6% indicated it was “not at all” challenging to use taste assessment tools.

### *2*^*nd*^*theme: assessment of clinicians’ experience with educational materials to learn about taste alterations*

The second theme of questions aims to assess participants’ experience with educational materials to learn about taste and altered taste function. Participants were asked several questions about access to educational materials and supportive information (e.g., websites, professional societies, and clinician training programs). The central theme of the questions was focused on where they could find information to learn about taste alterations and what they found most useful.

#### The gaps in the availability and the accessibility of educational materials

Participants were asked if they had access to adequate information to help patients manage their taste problems. Over half of the participants (58%) reported that they do not have access. Those who reported not having access indicated this was due to limited availability of resources and not knowing where and how to find them. For example, one participant mentioned, “There is no objective tool to actually understand the patient’s problem. I also don’t know how I would address the problem if I found it”. Additionally, some participants described that they did not receive adequate education and training on taste changes. One participant explains, “I have not received education on taste changes besides, radiation affects the taste buds. I was unaware of any potential treatment and am excited at the possibility.” When participants were asked how challenging it is to access educational material, 60% indicated it is at least “somewhat” challenging, with 22% reporting that it is “a little bit” challenging. However, 18% reported that it is “not at all” challenging to access educational materials.

**Fig. 2:**
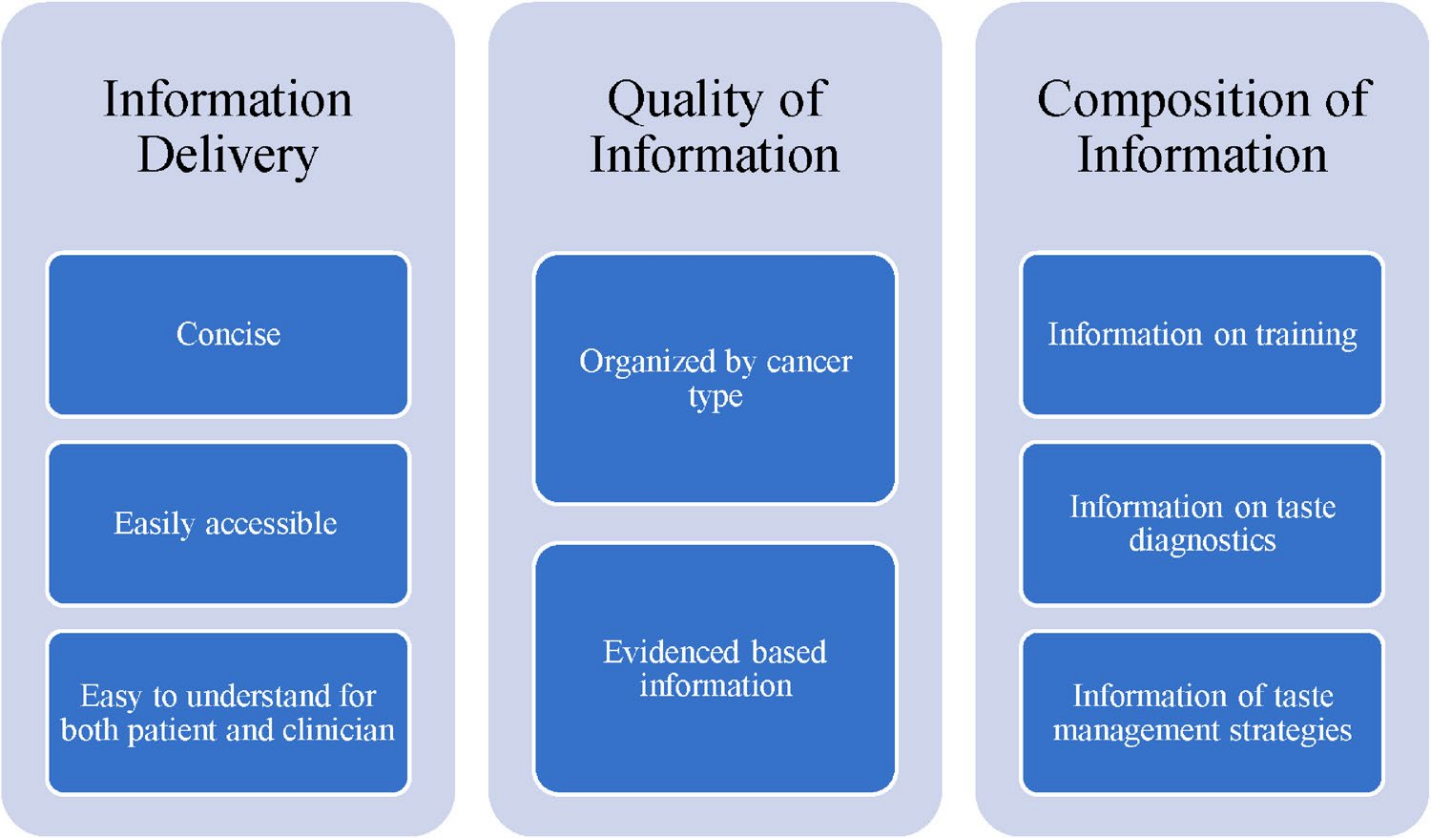
Participants describe important criteria to consider upon developing educational materials to facilitate learning about taste alterations

In an open-ended format, participants were asked to comment on what they thought would be potential options or platforms that would be useful for clinicians to learn about taste alterations and what should be included in these materials. In Fig. 1, we summarize four potential options suggested by participants, including materials provided through professional societies, dedicated programs or groups, a list of specialists and institutes for referring patients, and printable handouts and guides. Participants were asked to comment on the most important criteria that new educational materials should consist of to enable learning about taste alterations. Excerpts from participants’ responses regarding important criteria were categorized into three themes, information delivery, quality of information, and composition of information, shown in Fig. 2.

## Discussion

The present study examines clinicians’ current knowledge and understanding of taste alterations and taste-related terminologies, including taste disorder classifications and methods used in assessing taste function. The present study provides evidence that many clinicians remain unfamiliar with differences between taste and flavor, with less than a quarter of the participants selecting the correct definitions for both taste and flavor. These findings support prior work, in which dietitians and other oncology professionals describe the use of taste and flavor as being interchangeable [24, 25]. We expected that most participants would be unable to define different classifications of taste disorders; yet, in the present study, most participants correctly defined almost all the types of classifications of taste disorders. It is possible that participants were able to guess the definitions through the process of elimination, as the question format was a matching task. Taken together, this study, along with others [7, 24, 25, 29], suggests that for clinicians providing care for cancer patients, there is a knowledge gap regarding taste, flavor, and classifications of different taste disorders. Terminology that is confusing and misleading, and in this case differs from the common usage of the words, is thought to be one barrier to providing adequate care [7, 24, 25, 29], resulting in frustration for both clinicians and patients. This can make it challenging and difficult to communicate, diagnose, and treat patients, including whether their symptoms are only taste-related or include other systems involved with smell, flavor, and food hedonics [18, 25].

There are a variety of methods to evaluate taste function in clinics [30, 31] and in research settings [16, 18, 31–34]. Here, we reported that roughly half of the participants believe that taste assessment tools are somewhat challenging. Within the cancer population, self-assessment through questionnaires is the most common [25]. This trend is reflected in the present study with most participants reporting using self-reported questionnaires followed by informal interviewing, with only a quarter of the participants indicated to be using psychophysical methods in diagnosing dysgeusia. Even though self-assessment is convenient and easily accessible, most questionnaires do not differentiate between taste and flavor, raising concerns as to whether these questions accurately capture patient experiences [34, 35]. As an example, changes in retronasal olfaction are often diagnosed as “taste loss” or dysgeusia in instances that do not utilize a psychophysical method for diagnosing patients.

Moreover, differing methods of assessing taste loss results has made it challenging to summarize and compare findings within the published literature, resulting in wide variations in incidence and severity of taste loss among cancer patients [2, 36, 37]. Development of a validated and standardized assessment tool that is easy to administer will help to reduce the disparity in the use of different taste assessment tools among clinicians and may lead to better estimates of taste function and recovery in research and clinical studies. In the recent event of COVID-19, significant efforts have been made in developing questionnaires to assess both the quantity and quality of self-reported perception of chemosensory modalities in smell, taste, and chemesthesis (oral irritation). One such questionnaire is the GCCR questionnaire (Global Consortium for Chemosensory Research), which measures the self-reported smell, taste, and chemesthesis functions in addition to assessing the presence of nasal blockage and COVID-19. This questionnaire was developed by a group of chemosensory experts using a crowdsourced approach that would be a fast and easy-to-administer tool to measure the self-reported taste disorders of cancer patients in a clinical setting. It is anticipated that the high prevalence of loss of taste and smell among those suffering from a COVID-19 infection will increase the awareness of the importance of taste and smell and allow for more funds available to support research on the chemosenses.

We further identified that clinicians report not having adequate access to training or educational materials to learn about taste alterations and taste management strategies. Over half of the participants reported not having access to adequate information about taste alterations. This could be demonstrated by the observed confusion between the classification of taste and flavor, leading to confusion between patients and clinicians, and can become challenging when assessing dysgeusia. These findings support previous studies that emphasize the need for standardized terminology and validated taste assessment methods that are appropriate for clinical settings, along with accessible educational materials [24, 25]. For example, Boltong and colleagues (2011) noted that of the 89 medical entries examined, only 6 oncology clinicians referred to the terms of taste to clearly mean the sense of taste (i.e., sweet, sour, bitter, salty, and umami), whereas most referred elements of flavor and hedonics such as smell or touch, liking, appetite, or cognitive processing [25].

From the present study and prior work, clinicians report inadequate training and access to educational material. Belqaid and colleagues (2018) report that healthcare professionals’ involvement in supporting cancer patients to manage taste and smell alterations is limited but emphasize that the better involvement of clinicians has the potential to influence the patients for the adjustment to taste and smell alterations [16]. Based on the current study results, participants suggested a specific website for clinicians to provide information on taste, flavor, taste disorders, symptoms, and strategies that may help patients to manage their taste problems [25]. Increased education may facilitate improved communication between clinician and patient and could facilitate a feeling of support. Providing support for taste loss could improve patients’ quality of life since otherwise they may adjust their lives not to trust their senses [16]. The improvement in diagnosing and supporting cancer patients experiencing taste loss may help to address other co-occurring nutrition impact symptoms such as loss of appetite, impaired food enjoyment, altered food intake, early satiety, depression, and anxiety [10, 12, 18, 37–39]. Therefore, taste-related support could help patients’ nutritional status by improving their food intake and appetite. Overall, improving clinician training and knowledge regarding taste loss and reducing the communication barrier regarding taste will help patients feel supported and may lead to an improvement in their nutritional status and quality of life [40, 41].

For this study, the aim was to estimate clinicians’ knowledge of dysgeusia, which relied on an online survey. One limitation of this study is the use of multiple-choice and matching questions, which inherently lends itself to the risk of guessing and through the process of elimination. This limitation results in the possibility of overestimating the education and familiarity in this study. This study revealed the gaps in the availability and accessibility of educational materials to learn about taste alterations and brought up potential ways for such educational materials could be developed. This information could directly be used in developing educational materials specifically about taste alterations and may help to bring awareness of the lack of support that clinicians have about treating patients with taste alterations.

## Conclusion

There is growing evidence supporting the consequence of taste loss and its negative impact on health. The present study highlights clinicians’ knowledge and experience in supporting and managing cancer patients’ taste problems. Based on our findings and others, there is confusion regarding the vocabulary for taste and related terminology and unfamiliarity with taste assessment. Here, we conclude that clinicians lack adequate access to educational material, which differs from previous viewpoints suggesting this was due to taste symptoms not being life-threatening. The development of educational material and assessment tools that are easy to use, validated, and standardized will help to reduce barriers for clinicians and help to improve patient care. Supporting cancer patients for their taste symptoms may have profound clinical outcomes, such as improving food involvement, nutritional intake, and overall quality of life. This work demonstrates the need to improve training for clinicians, with a need to develop clinical assessment tools for clinical settings, and empirically evaluate treatments and management strategies for addressing cancer patients’ taste symptoms. While the current study focuses on taste symptoms, in order to provide comprehensive clinical support to patients, it is critical to consider olfactory function in addition to other sensory inputs (e.g., chemesthesis). Addressing these limitations will help to increase the resources for clinicians to support their patients, helping to improve patient outcomes and quality of life.

## Data Availability

The authors will make the data available upon request.
